# Porcine Reproductive and Respiratory Syndrome Virus strains with Higher Virulence Cause Marked Protein Profile Changes in MARC-145 Cells

**DOI:** 10.1038/s41598-018-32984-0

**Published:** 2018-10-09

**Authors:** Zhi Chen, Shaoning Liu, Shujin Zhang, Yuyu Zhang, Jiang Yu, Wenbo Sun, Lei Chen, Yijun Du, Jinbao Wang, Yubao Li, Jiaqiang Wu

**Affiliations:** 1 Shandong Key Lab of Animal Disease Control and Breeding, Shandong Academy of Agricultural Sciences, Jinan, 250100 China; 2 College of Life Sciences, Shandong Normal University, Jinan, 250014 China; 3 Shandong Institute of Veterinary Drug Quality Inspection, Jinan, 250022 China; 4 College of Agronomy, Liaocheng University, Liaocheng, 252000 China

## Abstract

Porcine reproductive and respiratory syndrome is an infectious disease that causes serious economic losses to the swine industry worldwide. To better understand the pathogenesis of the porcine reproductive and respiratory syndrome virus (PRRSV), three PRRSV strains with different molecular markers and virulence were used to infect MARC-145 cells. A total of 1804 proteins were identified, and 233 altered proteins and 72 signaling pathways involved in the proteomic profiling of virus-infected MARC-145 cells increased with the virulence of the PRRSV strain. The three types of viral strains shared a common pathway—the electron transport reaction in mitochondria—in the infected-MARC-145 cells. Moreover, the antisense pathway was the most variable of all significant signaling pathways for the highly virulent SX-1 strain, indicating that this unique pathway may be connected to the high virulence of the SX-1 strain. Our study is the first attempt to provide a proteome profile of MARC-145 cells infected with PRRSV strains with different virulence, and these findings will facilitate a deep understanding of the interactions between this virus and its host.

## Introduction

The porcine reproductive and respiratory syndrome virus (PRRSV), the causative agent of porcine reproductive and respiratory syndrome (PRRS), is an important pathogen in the swine industry, and it causes reproductive failure in pregnant sows and respiratory disorders in pigs of all ages^1^. As a member of the *Arteriviridae* family, PRRSV is an enveloped, linear, single positive-stranded RNA virus, and it is similar to the equine arteritis virus, lactate dehydrogenase-elevating virus, and simian hemorrhagic fever virus^2^. PRRSV can be classified into two distinct genotypes: type 1 (European type) and type 2 (North American type)^3,4^. *In vivo*, this virus mainly infects pulmonary alveolar macrophages (PAMs)^5–7^, whereas *in vitro*, MARC-145 cells are a good platform to study viral replication, pathogenesis, and host response^8–11^.

In life cycle of cells, there are some natural antisense transcripts (NATs), which are reverse complementarity with mRNAs^12^. These NATs, also named antisense RNAs or natural regulatory RNAs, are small molecules and non-coding RNAs, mediating regulation and generally inhibiting mRNA transcription and/or translation or inducing their rapid degradation^13,14^. Signals regarding antisense RNA transcription and regulation were named the antisense pathway, in which PSF (Polypyrimidine tract-binding protein-associated-splicing factor), P54 (Non-POU domain-containing octamer-binding protein) and MATR3 (matrin-3) are major proteins^15–17^. PSF is a DNA- and RNA- binding protein, involved in regulation of signal-induced alternative splicing and homologous DNA pairing^18^. The PSF-P54 heterodimer associated with MATR3 may play a role in nuclear retention of defective RNAs^19^. PSF also binds to and represses gene promoter region, such as CTBP1, P53, SMAD3 and P21^20,21^.

To explore the cellular mechanisms of PRRSV infection, mRNA expression has been analyzed at both molecular and cellular levels^11,22^. However, mRNA abundance is not always consistent with protein expression levels^23^. In recent years, proteomics analysis has been used to identify cellular protein expression profiles related to PRRSV infection^24–26^. Most reports of the proteomics analysis of PRRSV have described two-dimensional electrophoresis and mass spectrometry (MS) approaches^24,25,27^. In this study, we performed isobaric tags for relative and absolute quantitation (iTRAQ) labeling coupled with 2D/LC (liquid chromatography)–MS/MS analysis to detect the comparative protein profile of MARC-145 cells infected with different PRRSV strains^28^. Three differential virulent strains were used in this study, which are all belong to genotypes: type 2. SX-1 was a highly virulent strain which characterized with a 30aa deletion in nonstructural protein 2 (Nsp2) and isolated from domestic pigs in 2008^29^. The moderate virulent ZCYZ strain with a 54aa deletion in Nsp2 was isolated from hybrid wild boars in 2009^30^. The SD1 strain was a classic PRRSV strain with no deletion in Nsp2 which was mild virulent to pigs and isolated from domestic pigs in 2004^31^. This new method provided novel information regarding the proteomics of MARC-145 cells infected with different PRRSV strains.

## Experimental Procedures

### Cells and Viruses

MARC-145 cells were obtained from Shandong Key Lab of Animal Disease Control and Breeding. PAMs were harvested from 6-week-old clinically healthy piglets that were free of PRRSV, porcine circovirus, and *Mycoplasma* spp. PAMs were isolated, cultured, and infected as described preciously^32^. The PRRSV SD1 strain with no deletion in nonstructural protein 2 (Nsp2) was isolated from domestic pigs in 2004^31^, and the SX-1 strain with a 30aa deletion in Nsp2 was isolated from domestic pigs in 2008^29^. The virulence of SX-1 is high in pigs, as verified by animal infection experiments^29^. The ZCYZ strain with a 54aa deletion in Nsp2 was isolated from hybrid wild boars in 2009^30^. The virulences of the SX-1, ZCYZ, and SD1 strains are high, moderate, and mild, respectively, in pigs, as verified by animal infection experiments^29–31^.

### Sample Preparation, Digestion, and Labeling with iTRAQ Reagents

MARC-145 cells were incubated at 37 °C in 5% CO_2_ in DMEM medium (Gibco, Invitrogen, CA) supplemented with 8% fetal bovine serum (Fisher Scientific, Pittsburgh, PA). Cells were inoculated with MOI = 0.1 of the PRRSV SX-1, ZCYZ, or SD1 strain.

After being resuspended, sonicated, and quantified, cell samples were cysteines-blocked and digested with trypsin gold, according to the iTRAQ protocol (Applied Biosystems). The control cells were labeled with iTRAQ tag 118, and the three samples (SX-1-infected, ZCYZ-infected, and SD1-infected cells) were labeled with tags 113, 115, and 121, respectively. The labeled samples were then mixed prior to online 2D LC–MS/MS analysis.

### LC-ESI-MS/MS Analysis and Data Analysis

After fractionation through strong cationic exchange using the Shimadzu LC-20AB HPLC pump system, the fraction was resuspended and centrifuged. The peptides were subjected to nanoelectrospray ionization followed by tandem mass spectrometry (MS/MS) in an LTQ Orbitrap Velos (Thermo Fisher Scientific, Bremen, Germany) coupled online to the HPLC. For MS scans, the m/z (mass–charge ratio) scan range was 350–2000 Da.

Relative quantification and protein identification were performed using ProteinPilot^TM^ software 4.0.8085 with the Paragon algorithm (version 4.0.0.0) as a search engine. Each MS/MS spectrum was searched against a database of primate sequences (NCBInr, taxid9443). All identified proteins were grouped using the ProGroup algorithm (ABI) to minimize redundancy. The bias correction and background correction options were executed.

### Gene Ontology, Pathway Analysis, and Protein Signal Network Construction

Protein center software was used to analyze the functional distribution of these identified proteins. Pathway analysis was used to identify significant pathways of the differential genes according to KEGG, BioCarta, and Reatome. Fisher’s exact test and the χ^2^ test were used to select the significant pathways, and the threshold of significance was defined by *P*-values and false discovery rate (FDR).

To elucidate the differential protein–protein interactions and the signal transduction during PRRSV infection, signal networks were constructed to analyze the protein networks of MARC-145 cells infected by three PRRSV strains with different virulence. Each differentially expressed protein was analyzed and integrated into the network to elucidate the interactions.

### Confirmation of Proteomic Data in MARC-145 Cells and PAMs

Western blot and quantitative real-time polymerase chain reaction (PCR) were simultaneously performed in MARC-145 cells and PAMs to confirm proteomic data. An equivalent amount of protein was separated using 12% (w/v) sodium dodecyl sulfate–polyacrylamide gel electrophoresis. The fractionated proteins were then transferred electrophoretically to a PVDF membrane (Millipore, Bedford, MA) and blocked with TBS-T containing 5% bovine serum albumin at 4 °C overnight. The membranes were stained with goat anti-PSF polyclonal antibody (Santa Cruz, CA) at 1:200 dilutions, goat anti-annexin A2 (ANXA2) polyclonal antibody (Santa Cruz, CA) at 1:200 dilutions, and mouse anti-β-actin monoclonal antibody (Santa Cruz, CA) at 1:200 dilutions. After incubation at 37 °C for 1 h, immunoreactive protein bands were visualized with a chemiluminescence subtract using the ECL plus Western blot detection system (Kodak, NY). The quantification of protein blots was performed using Photoshop CS5 (Adobe, San Jose).

Total cellular RNA was extracted from MARC-145 cells or PAMs using Trizol reagent (Invitrogen, Canada) according to the manufacturer’s protocol. The quantification of RNA was performed with a Nanodrop 2000 (Thermo Scientific, Wilmington, DE USA), and 1 μg of total RNA was reverse-transcribed using the PrimeScript RT reagent kit (TaKaRa, China). cDNA was amplified using the SYBR Premix EX Taq II (TaKaRa, China). The primers used for the amplification of different target cDNAs are listed in Table 1. Quantification of the differences between the groups was performed using the 2^−ΔΔCt^ method. β-actin was used as the normalizing gene to compensate for potential differences in cDNA amounts.

**Table 1:** Primers used in real-time PCR.

Gene	Sequence
PAM β-actin	F: TCTGGCACCACACCTTCT R: GATCTGGGTCATCTTCTCAC
PAM PSF	F: TTGTTGGGAATCTACCTG R: GAACCCGAAGCTGTCTA
PAM ANXA2	F: ATCATGGTCTCCCGCAGTG R: AGTCGCCCTTGGTGTCTT
MARC-145 β-actin	F: CGGGAAATCGTGCGTGAC R: GCCCAGGAAGGAAGGTTG
MARC-145 PSF	F: TCGGTTGTTTGTTGGGAATC R: AAGCGAACTCGAAGCTGTCTA
MARC-145 ANXA2	F: TGACCAACCGCAGCAATG R: GAGCAGGTGTCTTCAATAGGC

### Gene Silencing with siRNA

MARC-145 cells grown to 60–70% confluence in 6-well cell culture plates were transfected with PSF siRNA, using Lipofectamine RNAiMAX (Invitrogen, Carlsbad, CA). Briefly, 60 pmol of siRNA was diluted in 250 μL of serum-free OptiMEM medium (Invitrogen, Carlsbad, CA), and 5 μL of Lipofectamine RNAiMAX was diluted in 250 μL of OptiMEM. After incubation for 5 min at room temperature, the diluted RNA and Lipofectamine RNAiMAX were combined and incubated again for 20 min at room temperature. Then, 500 μL of the mixture was added dropwise to each well. The plates were incubated at 37 °C in a CO_2_ incubator for 8 h, and the mixtures were replaced with fresh complete medium and incubated for an additional 40 h before the silencing efficiency was measured using immunoblotting analysis. Scrambled siRNA was used as a negative control.

## Results

### PRRSV-Infected MARC-145 Cells and iTRAQ Analysis

After inoculation with PRRSV SX-1, ZCYZ, or SD1 strains of MOI = 0.1, the MARC-145 cells were collected at 48 hpi and used for the iTRAQ experiment. Uninfected MARC-145 cells served as a mock control. After digestion, quantification and labeling were performed according to the iTRAQ protocol (Applied Biosystems)^33^, and the 4-plex iTRAQ experiments were conducted.

### Gene Ontology and Pathway and Protein Signal Network Analyses

**Figure 1:**
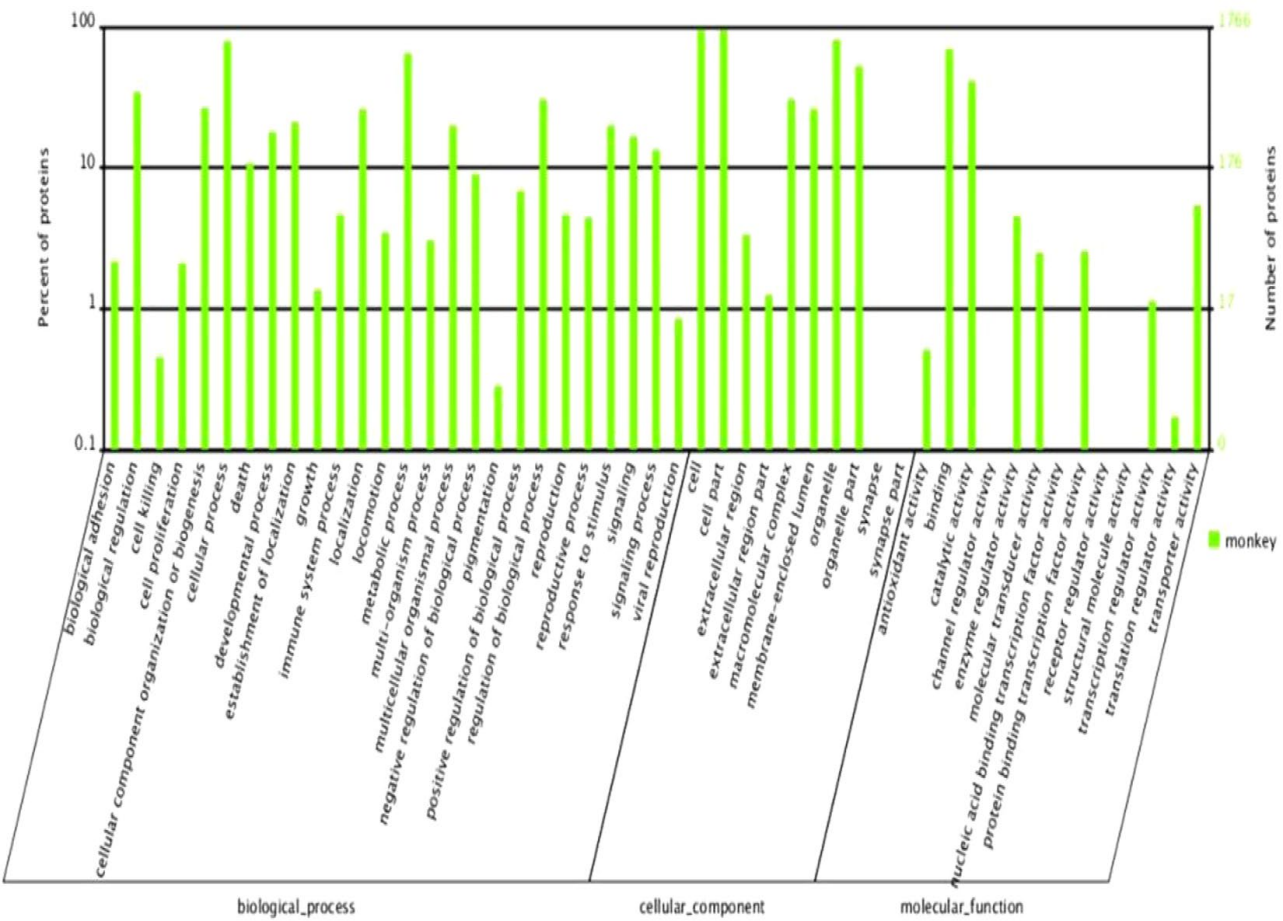
Results of gene ontology (GO). Three main types of annotations were obtained from the gene ontology consortium website: cellular components, molecular function, and biological distribution.

Functional annotation of the 1804 identified proteins was initially performed using the Protein Center software. A *P*-value of <0.05 and an FDR of <0.05 in a two-sided Fisher’s exact test were selected as significant criteria. Three main types of annotations were obtained from the gene ontology consortium website: cellular components, molecular function, and biological distribution (Fig. 1). A number of the identified proteins were involved in cellular and metabolic processes and biological regulation. Moreover, the molecular function of the identified proteins was associated with binding and catalytic activity. A total of 233 differentially expressed proteins were discovered in this study. Furthermore, 105 proteins were identified as differentially expressed in Group I (SX-1 vs. MARC-145), including 46 upregulated proteins and 59 downregulated proteins (Table 2); 84 proteins were identified as differentially expressed in Group II (ZCYZ vs. MARC-145), including 59 upregulated proteins and 25 downregulated proteins (Table 3); and 44 proteins were identified as differentially expressed in Group III (SD1 vs. MARC-145), including 25 upregulated proteins and 19 downregulated proteins (Table 4). In the three groups, the upregulated and downregulated differential proteins involved in significant pathways were sorted through the enrichment of signaling pathway categories (Fig. 1).

**Table 2:** Group I (SX-1 vs MARC-145).

Query	Genesymbol	accession	regulation
gi|109078430	LMNB1	P20700	up
gi|109083078	PCK2	Q16822	down
gi|109087706	FLNB	O75369	up
gi|109128138	ALDOA	P04075	up
gi|114560493	VAV3	Q9UKW4	down
gi|114577891	GFPT1	Q06210	down
gi|114582378	HSPD1	P10809	up
gi|114608498	SYNCRIP	O60506	up
gi|114614114	MDH2	P40926	up
gi|114638643	SF3B2	Q13435	up
gi|114646614	HSP90AA1	P07900	down
gi|114662315	LOC100133770	Q96QK1	down
gi|114674909	KHSRP	Q92945	up
gi|119573383	LMNA	P02545	up
gi|119589784	LMNB2	Q03252	up
gi|119606358	ITGB1	P05556	up
gi|148727343	MDH1	P40925	down
gi|158256818	COPG	Q9Y678	down
gi|169402708	RALY	Q9UKM9	up
gi|17986283	TUBA1A	Q71U36	down
gi|194386548	MMS19	Q96T76	down
gi|2183299	ALDH1A1	P00352	down
gi|22219421	ANXA6	P08133	down
gi|250127	RPSA	P08865	down
gi|281183052	FLNA	P21333	up
gi|281183276	GART	P22102	down
gi|292059	HSPA8	P11142	up
gi|296192884	MATR3	P43243	up
gi|296197135	SOS1	Q07889	up
gi|296205845	NCL	P19338	up
gi|296208146	PGM1	P36871	down
gi|296212003	MYL6B	P14649	up
gi|296219568	RPS20	P60866	up
gi|296229779	PRDX6	P30041	down
gi|296230808	HNRNPU	Q00839	down
gi|296231979	CCT8	P50990	down
gi|297263416	CKAP4	Q07065	up
gi|297266457	IMMT	Q16891	up
gi|297267662	AHNAK	Q09666	down
gi|297271343	IARS	P41252	up
gi|297271540	MYO1C	O00159	down
gi|297282358	UBR5	O95071	down
gi|297284439	AARS	P49588	down
gi|297285873	SMARCC1	Q92922	up
gi|297293189	HADH	Q16836	up
gi|297302073	MKI67	P46013	up
gi|297305113	G6PD	P11413	down
gi|297716361	SP1	P08047	down
gi|302565376	C14orf142	Q9BXV9	down
gi|306482649	RPLP0	P05388	down
gi|306875	HNRNPCL1	O60812	up
gi|307133695	EEF1G	P26641	down
gi|310923118	TXN	P10599	up
gi|3126878	HNRNPM	P52272	down
gi|32189394	ATP5A1	P25705	down
gi|33112236	CAPN1	P07384	down
gi|3668141	RSL1D1	O76021	up
gi|36796	TCP1	P17987	down
gi|407308	P54	Q15233	up
gi|4235275	TLN1	Q9Y490	up
gi|44771201	INTS5	Q6P9B9	down
gi|4504523	HSPE1	P61604	up
gi|4505941	POLR2B	P30876	down
gi|4506243	PTBP1	P26599	up
gi|4506623	RPL27	P61353	up
gi|4758012	CLTC	Q00610	down
gi|4758302	ERH	P84090	up
gi|48146175	EIF3E	P60228	down
gi|48146275	SOS2	Q07890	down
gi|4826998	PSF	P23246	up
gi|499158	ACAT1	P24752	up
gi|5006602	ILF3	Q12906	up
gi|5031973	PDIA6	Q15084	down
gi|5032087	SF3A1	Q15459	up
gi|5107666	MTOR	P42345	down
gi|52545896	HNRNPUL2	Q1KMD3	up
gi|52545934	XPO1	O14980	down
gi|542850	RBMX	P38159	up
gi|5453998	IPO7	O95373	down
gi|5803187	TALDO1	P37837	down
gi|5803225	YWHAE	P62258	up
gi|62087384	FUS	P35637	up
gi|693937	UBR5	O95071	up
gi|70980549	PDCD11	Q14690	down
gi|71891685	CAND1	Q86VP6	down
gi|73620030	C9orf64	Q5T6V5	down
gi|73909156	ANXA2	P07355	down
gi|7417372	HABP4	Q5JVS0	down
gi|75040155	GBP1	P32455	up
gi|75075786	ARL6IP5	O75915	down
gi|75075845	VIM	P08670	up
gi|82400267	AKR1C3	P42330	down
gi|8392875	C16orf80	Q9Y6A4	down
gi|89365957	EIF3A	Q14152	down
gi|90075022	CCT3	P49368	down
gi|90075818	HSP90AA1	P07900	down
gi|90076298	WARS	P23381	up
gi|90076340	ECHS1	P30084	down
gi|90076382	SSB	P05455	down
gi|90077474	RARS	P54136	down
gi|90080277	RPS3	P23396	down
gi|90083957	HNRNPA2B1	P22626	down
gi|94429050	SEC22B	O75396	down
gi|951338	CSE1L	P55060	down
gi|97536594	MDH1	P40925	down

**Table 3:** Group II (ZCYZ vs MARC-145).

Query	genesymbol	accession	regulation
gi|109003906	NASP	P49321	down
gi|109067156	IGF2BP3	O00425	up
gi|109094347	ACO2	Q99798	up
gi|109096866	KRT18	P05783	down
gi|109102941	FLNC	Q14315	up
gi|114557920	RPL5	P46777	up
gi|114560493	VAV3	Q9UKW4	up
gi|114572703	EPRS	P07814	up
gi|114607013	RPL10A	P62906	up
gi|114608498	SYNCRIP	O60506	up
gi|114621209	YWHAZ	P63104	up
gi|114624610	TOMM5	Q8N4H5	up
gi|119593144	RPL10	P27635	down
gi|119593252	RPL18A	Q02543	down
gi|119600189	RPL24	P83731	down
gi|119628097	HERC4	Q5GLZ8	down
gi|13543551	PSMA1	P25786	up
gi|16507237	HSPA2	P54652	up
gi|17426164	FLNB	O75369	down
gi|17986283	TUBA1A	Q71U36	down
gi|194380122	DNM1L	O00429	up
gi|19923193	PPP5C	P53041	up
gi|217272851	P4HA1	P13674	up
gi|2183299	ALDH1A1	P00352	up
gi|281182974	PDIA3	P30101	up
gi|281183052	FLNB	O75369	up
gi|292059	HSPA8	P11142	up
gi|296205836	PSMD1	Q99460	up
gi|296208633	RTCD1	O00442	up
gi|296219568	RPS20	P60866	up
gi|296230808	HNRNPU	Q00839	up
gi|297262690	CS	O75390	up
gi|297264568	MYO1B	O43795	up
gi|297267548	PPP5C	P53041	up
gi|297271343	IARS	P41252	down
gi|297272588	NME2	P22392	up
gi|297274229	HSPA8	P11142	up
gi|297275803	EEF2	P13639	up
gi|297277187	PAFAH1B3	Q15102	up
gi|297280846	RPS25	P62851	down
gi|297293189	HADH	Q16836	up
gi|297664392	VAV3	Q9UKW4	down
gi|297671227	MANF	P55145	up
gi|297703839	DDX3X	O00571	down
gi|302565376	C14orf142	Q9BXV9	down
gi|306482641	GAPDH	P04406	up
gi|310923118	TXN	P10599	up
gi|3126878	HNRNPM	P52272	up
gi|32189394	ATP5A1	P25705	up
gi|4501891	FLNC	Q14315	down
gi|4502297	ATP5D	P30049	down
gi|4504281	HIST1H3A	P68431	up
gi|4506189	PSMA7	O14818	up
gi|4506623	RPL27	P61353	up
gi|4506699	RPS21	P63220	down
gi|4758302	ERH	P84090	up
gi|4758304	PDIA4	P13667	up
gi|48145985	PDCD5	O14737	down
gi|55728072	NAP1L4	Q99733	down
gi|55729123	NCSTN	Q92542	down
gi|55729581	ACSL3	O95573	up
gi|55824566	PSMB4	P28070	up
gi|5650709	GNA12	Q03113	up
gi|5729877	HSPA8	P11142	up
gi|5803225	YWHAE	P62258	down
gi|5821385	NUDT1	P36639	up
gi|6005854	PHB2	Q99623	down
gi|62896507	NPC2	P61916	up
gi|67969713	PSMA2	P25787	up
gi|67970515	PHB	P35232	down
gi|6912598	NT5C2	P49902	up
gi|736677	DLST	P36957	up
gi|73909156	ANXA2	P07355	down
gi|75040155	GBP1	P32455	up
gi|75056681	CYCS	P99999	up
gi|75075777	NDRG1	Q92597	up
gi|75766221	CLIC4	Q9Y696	down
gi|7669550	VCL	P18206	up
gi|7705425	MRPS17	Q9Y2R5	up
gi|90075940	AHCY	P23526	up
gi|90076298	WARS	P23381	up
gi|90077334	ACADVL	P49748	up
gi|90083957	HNRNPA2B1	P22626	up
gi|92859595	PELP1	Q8IZL8	down

**Table 4:** Group III (SD1 vs MARC-145).

Query	genesymbol	accession	regulation
gi|109003906	NASP	P49321	down
gi|109083078	PCK2	Q16822	down
gi|109087706	FLNC	Q14315	up
gi|109119169	FASN	P49327	down
gi|114603763	HNRNPAB	Q99729	up
gi|114624610	TOMM5	Q8N4H5	up
gi|114686445	DDX3X	O00571	down
gi|119573383	LMNA	P02545	up
gi|119589784	LMNB2	Q03252	up
gi|2463577	PRPF8	Q6P2Q9	down
gi|281183052	FLNA	P21333	up
gi|296205845	NCL	P19338	up
gi|296212003	MYL6B	P14649	up
gi|296219568	RPS20	P60866	up
gi|297266457	IMMT	Q16891	up
gi|297267662	AHNAK	Q09666	up
gi|297284439	AARS	P49588	down
gi|297292908	PDGFRA	P16234	down
gi|297293189	HADH	Q16836	up
gi|297295501	SPARC	P09486	down
gi|297302073	MKI67	P46013	up
gi|302565376	C14orf142	Q9BXV9	down
gi|32189394	ATP5A1	P25705	down
gi|44771201	INTS5	Q6P9B9	down
gi|4504523	HSPE1	P61604	up
gi|4505641	PCNA	P12004	up
gi|4505763	PGK1	P00558	up
gi|4505941	POLR2B	P30876	down
gi|4506623	RPL27	P61353	up
gi|45861372	DDX58	O95786	down
gi|498910	AIMP1	Q12904	up
gi|52545934	XPO1	O14980	down
gi|542850	RBMX	P38159	up
gi|55729123	NCSTN	Q92542	down
gi|5729877	HSPA8	P11142	up
gi|5821385	NUDT1	P36639	up
gi|62896507	NPC2	P61916	up
gi|693937	UBR5	O95071	up
gi|73909156	ANXA2	P07355	down
gi|7669550	VCL	P18206	up
gi|82400267	AKR1C3	P42330	down
gi|90075448	DDX3Y	O15523	down
gi|90076298	WARS	P23381	up
gi|90076506	PSAP	P07602	down

**Figure 2:**
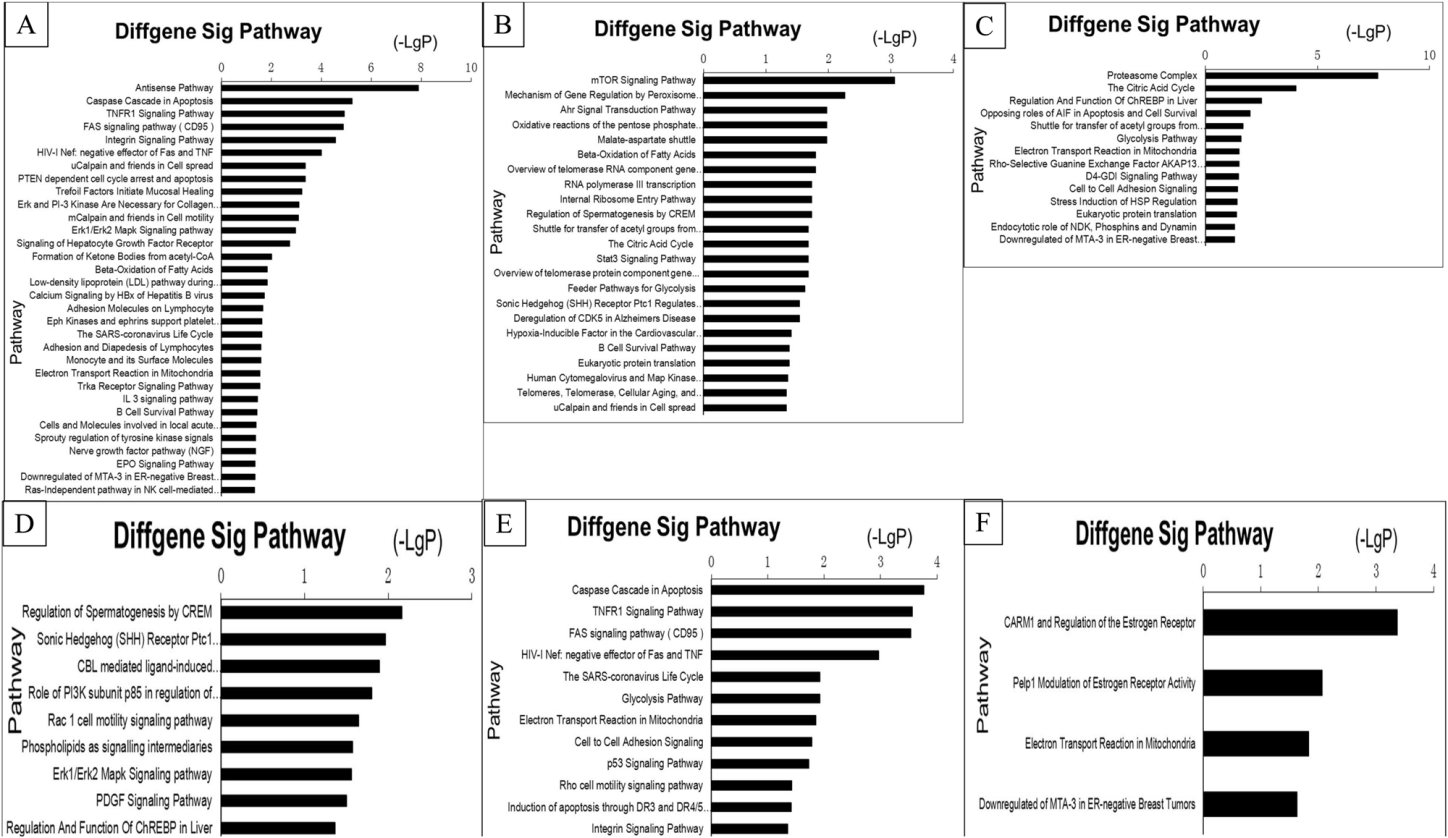
Enriched signaling pathway categories of differentially expressed proteins from three groups. (**A**) Significant signaling pathways of upregulated proteins in Group I. (**B**) Significant signaling pathways of downregulated proteins in Group I. (**C**) Significant signaling pathways of upregulated proteins in Group II. (**D**) Significant signaling pathways of downregulated proteins in Group II. (**E**) Significant signaling pathways of upregulated proteins in Group III. (**F**) Significant signaling pathways of downregulated proteins in Group III. ACADVL: very long-chain specific acyl-CoA dehydrogenase; ACAT1: acetyl-CoA acetyltransferase; ACO2: Aconitate hydratase; ACSL3: Long-chain-fatty-acid–CoA ligase 3; AHCY: Adenosylhomocysteinase; AHNAK: Neuroblast differentiation-associated protein; AIMP1: Aminoacyl tRNA synthase complex-interacting multifunctional protein 1; AKR1C3: Aldo-keto reductase family 1 member C3; ALDH1A1: retinal dehydrogenase 1; ALDOA: Fructose-bisphosphate aldolase A; ANXA6: Annexin A6; ARL6IP5: PRA1 family protein 3; ATP5A1: ATP synthase subunit alpha; ATP5D: ATP synthase subunit delta; C14orf142: EKC/KEOPS complex subunit; C16orf80: Cilia- and flagella-associated protein 20; C9orf64: Queuosine salvage protein; CAND1: Cullin-associated NEDD8-dissociated protein 1; CAPN1: Calpain-1 catalytic subunit; CCT3: T-complex protein 1 subunit gamma; CCT8: T-complex protein 1 subunit theta; CKAP4: Cytoskeleton-associated protein 4; CLIC4: Chloride intracellular channel protein 4; CLTC: Clathrin heavy chain 1; COPG: Coatomer subunit gamma; CS: Citrate synthase; CSE1L: Exportin-2; CYCS: Cytochrome c, somatic; DDX3X: ATP-dependent RNA helicase DDX3X; DDX3Y: ATP-dependent RNA helicase DDX3Y; DDX58: Probable ATP-dependent RNA helicase DDX58; DLST: Dihydrolipoyllysine-residue succinyltransferase component of 2-oxoglutarate dehydrogenase complex; DNM1L: Dynamin-1-like protein; ECHS1: Enoyl-CoA hydratase; EEF1G: Elongation factor 1-gamma; EEF2: Eukaryotic elongation factor 2 kinase; EIF3A: Eukaryotic translation initiation factor 3 subunit A; EIF3E: Eukaryotic translation initiation factor 3 subunit E; EPRS: Bifunctional glutamate/proline–tRNA ligase; ERH: Enhancer of rudimentary homolog; FASN: Fatty acid synthase; FLNA: Filamin-A; FLNB: Filamin-B; FLNC: Filamin-C; FUS: RNA-binding protein FUS; G6PD: Glucose-6-phosphate 1-dehydrogenase; GAPDH: lyceraldehyde-3-phosphate dehydrogenase; GART: Trifunctional purine biosynthetic protein adenosine-3; GBP1: Guanylate-binding protein 1; GFPT1:Glutamine–fructose-6-phosphate aminotransferase [isomerizing] 1; GNA12: Guanine nucleotide-binding protein subunit alpha-12; HABP4: Intracellular hyaluronan-binding protein 4; HADH: Hydroxyacyl-coenzyme A dehydrogenase; HERC4: Probable E3 ubiquitin-protein ligase HERC4; HIST1H3A: Histone H3.1; HNRNPA2B1: Heterogeneous nuclear ribonucleoproteins A2/B1; HNRNPAB: Heterogeneous nuclear ribonucleoprotein A/B; HNRNPCL1: Heterogeneous nuclear ribonucleoprotein C-like 1; HNRNPM: Heterogeneous nuclear ribonucleoprotein M; HNRNPU: Heterogeneous nuclear ribonucleoprotein U; HNRNPUL2: Heterogeneous nuclear ribonucleoprotein U-like protein 2; HSP90AA1: Heat shock protein HSP 90-alpha; HSPA2: Heat shock-related 70 kDa protein 2; HSPA8: Heat shock cognate 71 kDa protein; HSPD1: 60 kDa heat shock protein; HSPE1: 10 kDa heat shock protein IARS: Isoleucine–tRNA ligase; IGF2BP3: Insulin-like growth factor 2 mRNA-binding protein 3; ILF3: Interleukin enhancer-binding factor 3; IMMT: MICOS complex subunit MIC60; INTS5: Integrator complex subunit 5; IPO7: Importin-7; ITGB1: Integrin beta-1; KHSRP: Far upstream element-binding protein 2; KRT18: Keratin, type I cytoskeletal 18; LMNA: Prelamin-A/C; LMNB2: Lamin-B2; MANF: Mesencephalic astrocyte-derived neurotrophic factor; MATR3: Matrin-3; MDH1: Malate dehydrogenase; MDH2: Malate dehydrogenase; MKI67: Proliferation marker protein Ki-67; MMS19: MMS19 nucleotide excision repair protein homolog; MRPS17: 28S ribosomal protein S17; MTOR: Serine/threonine-protein kinase mTOR; MYL6B: Myosin light chain 6B; MYO1B: Unconventional myosin-Ib; MYO1C: Unconventional myosin-Ic; NAP1L4: Nucleosome assembly protein 1-like 4; NASP: Nuclear autoantigenic sperm protein; NCL: Nucleolin; NCSTN: Nicastrin; NDRG1: N-myc downstream-regulated gene 1 protein; NME2: Nucleoside diphosphate kinase B; NPC2: NPC intracellular cholesterol transporter 2; NT5C2: Cytosolic purine 5′-nucleotidase; NUDT1: 7,8-dihydro-8-oxoguanine triphosphatase; P4HA1: Prolyl 4-hydroxylase subunit alpha-1; P54: Non-POU domain-containing octamer-binding protein(NONO); PAFAH1B3: Platelet-activating factor acetylhydrolase IB subunit gamma; PCK2: Phosphoenolpyruvate carboxykinase [GTP]; PCNA: PCNA-associated factor; PDCD11: Protein RRP5 homolog; PDCD5: Programmed cell death protein 5; PDGFRA: Platelet-derived growth factor receptor alpha; PDIA3: Protein disulfide-isomerase A3; PDIA4: Protein disulfide-isomerase A4; PDIA6: Protein disulfide-isomerase A6; PELP1: Proline-, glutamic acid- and leucine-rich protein 1; PGK1: Phosphoglycerate kinase 1; PGM1: Phosphoglucomutase-1; PHB: Prohibitin; PHB2: Prohibitin-2; POLR2B: DNA-directed RNA polymerase II subunit RPB2; PPP5C: Serine/threonine-protein phosphatase 5; PRDX6: Peroxiredoxin-6; PRPF8: Pre-mRNA-processing-splicing factor 8; PSAP: Prosaposin; PSF: Splicing factor, proline- and glutamine-rich(SFPQ); PSMA1: Proteasome subunit alpha type-1; PSMA2: Proteasome subunit alpha type-2; PSMA7: Proteasome subunit alpha type-7; PSMB4: Proteasome subunit beta type-4; PSMD1: 26S proteasome non-ATPase regulatory subunit 1; PTBP1: Polypyrimidine tract-binding protein 1; RALY: RNA-binding protein Raly; RARS: Arginine-tRNA ligase; RBMX: RNA-binding motif protein, X chromosome; RPL10: 60S ribosomal protein L10; RPL10A: 60S ribosomal protein L10-1; RPL24: 60S ribosomal protein L24; RPL27: 60S ribosomal protein L27; RPL5: 60S ribosomal protein L5; RPLP0: 60S acidic ribosomal protein P0; RPS20: 40S ribosomal protein S20; RPS21: 40S ribosomal protein S21; RPS25: 40S ribosomal protein S25; RPS3: 40S ribosomal protein S3; RPSA: 30S ribosomal protein S1; RSL1D1: Ribosomal L1 domain-containing protein 1; RTCD1: RNA 3′-terminal phosphate cyclase; SEC. 22B: Vesicle-trafficking protein SEC. 22b; SF3A1: Splicing factor 3A subunit 1; SF3B2: Splicing factor 3B subunit 2; SMARCC1: WI/SNF-related matrix-associated actin-dependent regulator of chromatin subfamily C member 1; SOS1: Son of sevenless homolog 1; SOS2: Son of sevenless homolog 2; SP1: Transcription factor Sp1; SPARC: Basement-membrane protein 40; SSB: Lupus La protein; SYNCRIP: Heterogeneous nuclear ribonucleoprotein Q; TALDO1: Transaldolase; TCP1: T-complex protein 1 subunit alpha; TLN1: Talin-1; TOMM5: Mitochondrial import receptor subunit TOM5 homolog; TUBA1A: Tubulin alpha-1A chain; TXN: Thioredoxin; UBR5: E3 ubiquitin-protein ligase UBR5; VAV3: Guanine nucleotide exchange factor VAV3; VCL: Vinculin; VIM: Vimentin; WARS: Tryptophan–tRNA ligase; XPO1: Exportin-1; YWHAE: 14-3-3 protein epsilon; YWHAZ: 14-3-3 protein zeta/delta.

In Group I (SX-1 vs. MARC-145), the significant signaling pathways of upregulated proteins included the antisense pathway, caspase cascade in apoptosis, TNFR1 signaling pathway, FAS signaling pathway (CD95), integrin signaling pathway, HIV-1 Nef: negative effector of Fas and TNF, etc. (Fig. 2A). By contrast, the significant pathways of the downregulated proteins included the mTOR signaling pathway, mechanism of gene regulation by peroxisome proliferators via PPARa (alpha), Ahr signal transduction pathway, oxidative reactions of the pentose phosphate pathway, malate–aspartate shuttle, etc. (Fig. 2B). In Group II (ZCYZ vs. MARC-145), the significant pathways corresponding to the upregulated proteins included the proteasome complex, citric acid cycle, regulation and function of ChREBP in the liver, opposing roles of AIF in apoptosis and cell survival, shuttle for transfer of acetyl groups from mitochondria to the cytosol, etc. (Fig. 2C). The significant pathways of the downregulated proteins were the regulation of spermatogenesis by CREM, Sonic Hedgehog (SHH) Receptor Ptc1 regulates cell cycle, CBL-mediated ligand-induced downregulation of EGF receptors, role of the PI3K subunit p85 in the regulation of actin organization and cell migration, Rac 1 cell motility signaling pathway, etc. (Fig. 2D). In Group III (SD1 vs. MARC-145), the significant pathways corresponding to upregulated proteins included the caspase cascade in apoptosis, TNFR1 signaling pathway, FAS signaling pathway (CD95), HIV-I Nef: negative effector of Fas and TNF, SARS-coronavirus life cycle, and glycolysis pathway (Fig. 2E). The significant pathways of the downregulated proteins included CARM1 and regulation of the estrogen receptor, Pelp1 modulation of estrogen receptor activity, electron transport reaction in mitochondria, and downregulation of MTA-3 in ER-negative breast tumors (Fig. 2F).

**Figure 3:**
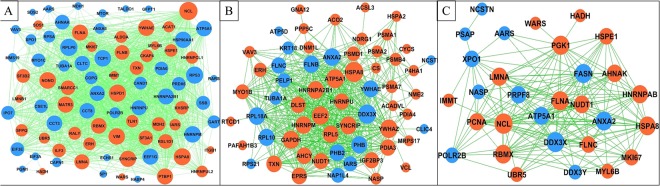
Signaling networks of differentially expressed proteins. (**A**) Signaling networks of differentially expressed proteins in Group I. (**B**) Signaling networks of differentially expressed proteins in Group II. (**C**) signaling networks of differentially expressed proteins in Group III. Upregulated proteins are shown in red, whereas downregulated proteins are shown in blue. Circle sizes represent the capacity of a protein to interact with other proteins, which is quantified in degrees. The greater degree a protein has, the more altered proteins interact with it. SFPQ and NONO are the synonyms of PSF and P54, respectively.

A signaling network was used to establish possible interactions among the differentially expressed proteins. In MARC-145 cells infected with SX-1, the most central proteins are NCL, RPLPO, HSPD1, RPS3, HSPA8, P54, HNRNPM, TCP1, VIM, HSP90AA1, MATR3, SYNCRIP, YMHAE, EEF1G, FLNA, HNRNPA2B1, PTBP1, ATP5A1, CCT3, RBMX, CCT8, ANXA2, CLTC, HNRNPU, LMNA, AHNAK, and FLNB (Fig. 3A). In MARC-145 cells infected with ZCYZ, the most central proteins included HSPA8, DDX3X, ATP5A1, YWHAE, EEF2, YWHAZ, GAPDH, HNRNPA2B1, SYNCRIP, ANXA2, HNRNPM, RPL18A, FLNB, and PHB (Fig. 3B). In MARC-145 cells infected with SD1, NCL, DDX3X, FASN, FLNA, HSPA8, PGK1, ANXA2, RBMX, ATP5A1, HNRNPAB, and LMNA exhibited as the most central proteins (Fig. 3C). These proteins tended to be more essential than noncentral proteins in the modular organization of the protein–protein interaction network.

### Confirmation of Proteomic Data

**Figure 4:**
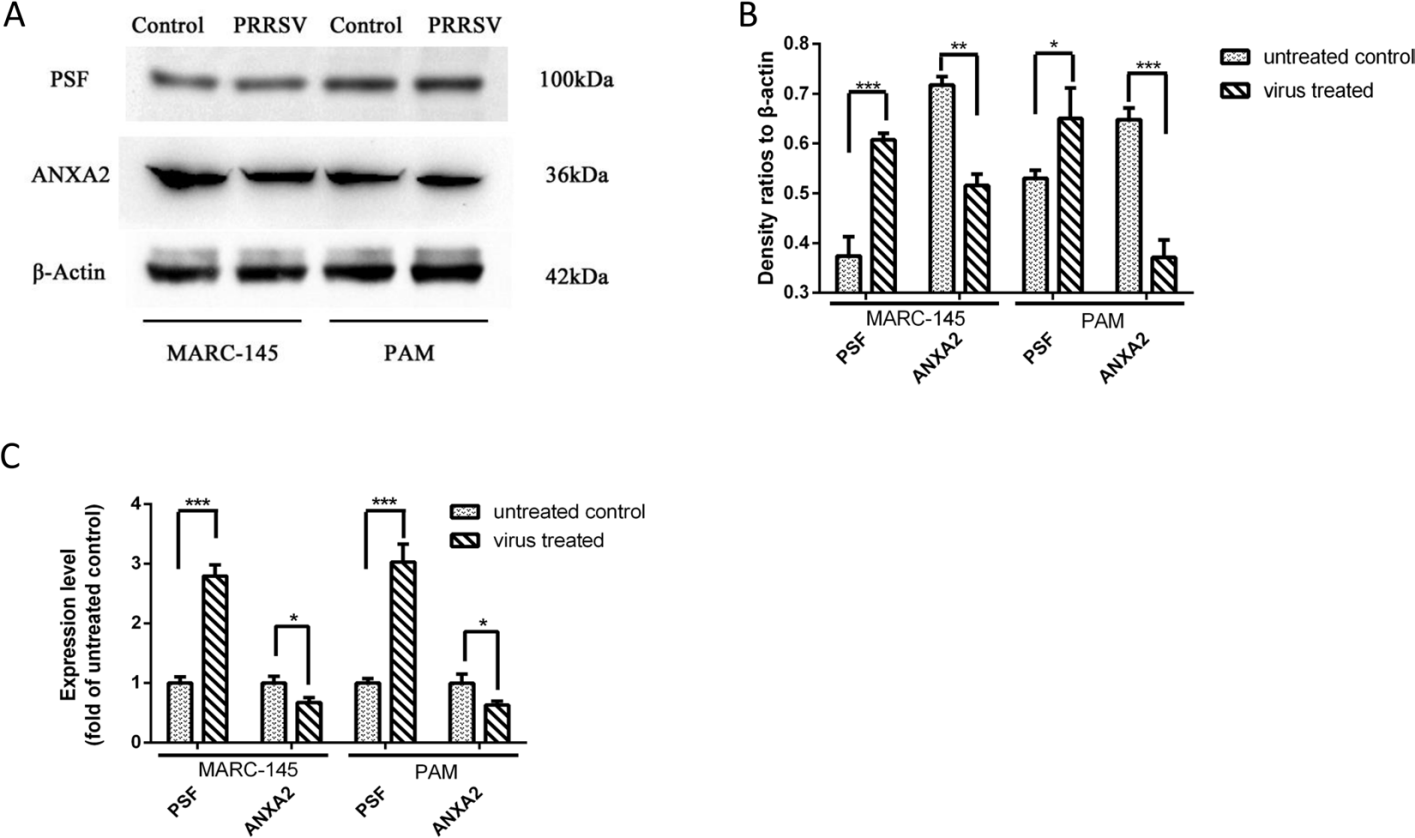
Confirmation of proteomic data by Western blot and real-time PCR. (**A**) MARC-145 cells and PAMs infected with SX-1 at 48 hpi were harvested and lysed; then, cell extracts were separated by SDS-PAGE and analyzed through immunoblotting with anti-HSPA8 and anti-ANXA2 antibodies. β-actin was used as a protein loading control. (**B**) Representative results are shown in a graph representing the density ratio to β-actin normalized to the control condition. (**C**) A graph of the quantified transcript levels of MARC-145 cells and PAMs infected with SX-1. Gene expression was quantified using real-time PCR and the comparative critical threshold (2^−ΔΔCt^) method. The β-actin gene was used as the endogenous reference. Three independent experiments were performed. The data from three independent trails are represented as mean ± SD. *t*-test; *p < 0.05; **p < 0.01; ***p < 0.001.

To validate the findings of differentially expressed proteins identified by the iTRAQ labeled LC–MS/MS system, PSF and ANXA2 were analyzed using Western blotting (Fig. 4A), and β-actin was used as an internal control. The results revealed that PSF was upregulated in both MARC-145 cells and PAMs infected with SX-1 at 48 hpi, whereas Annexin A2 was downregulated in both MARC-145 cells and PAMs infected with SX-1 at 48 hpi. Further quantitative analyses demonstrated that PSF and ANXA2 expression significantly differed between the PRRSV-infected and control groups (Fig. 4B). Similar results were obtained from the quantitative real-time PCR assay (Fig. 4C). Taken together, the Western blotting and quantitative real-time PCR results were consistent with those of the iTRAQ-coupled 2D LC–MS/MS analysis.

### Knockdown of Endogenous PSF Genes Decreases Replication of the PRRSV SX-1 Strain

**Figure 5:**
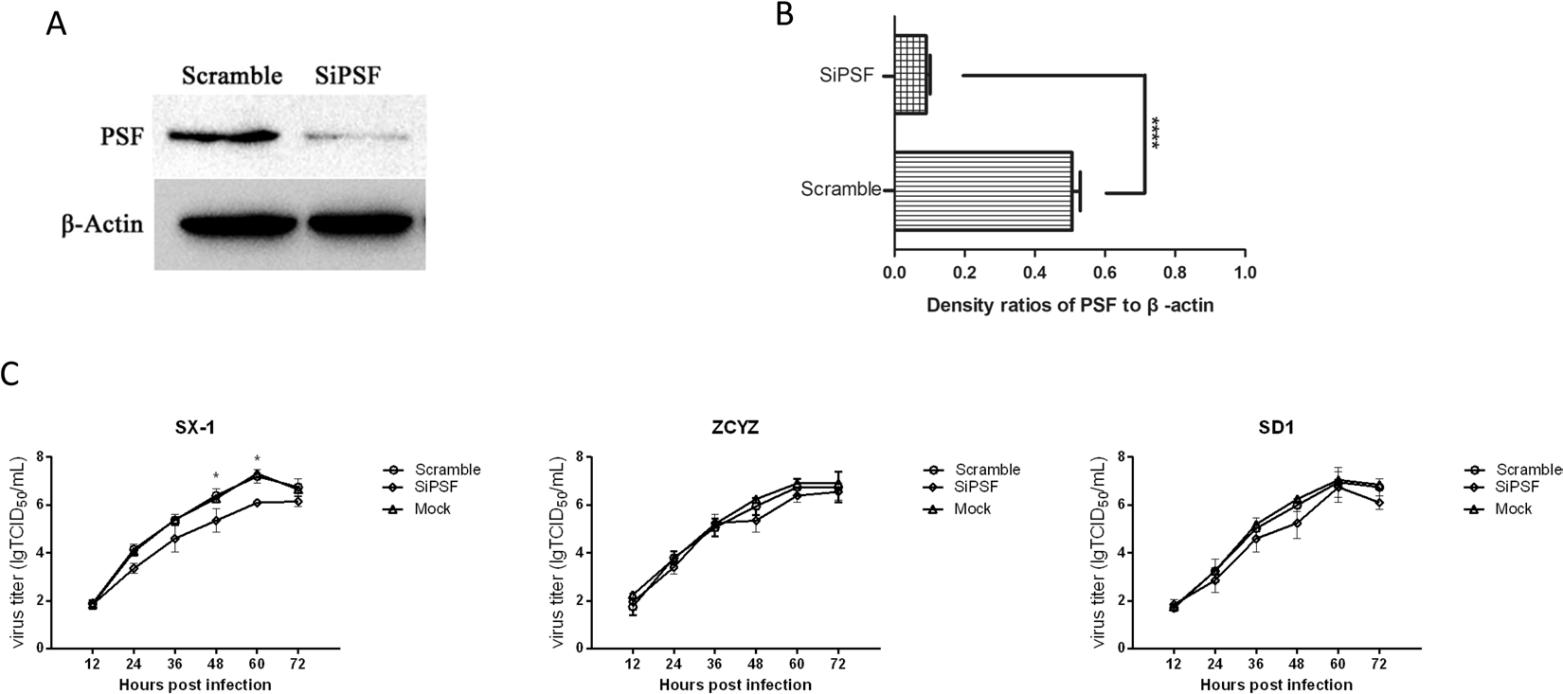
Knockdown of Endogenous PSF Genes Decreases Replication of the PRRSV SX-1 Strain. (**A**) Endogenous PSF protein expression was down-regulated by PSF siRNA (SiPSF). SiRNA was transfected by Lipofectamine RNAiMAX (Invitrogen, Carlsbad, CA) according to the manufacturer’s instruction. Scrambled siRNA was used as a negative control. After transfected 48 h, cells were harvested, and western blotting was performed. (**B**) Graphical representation of *t*-test of the ratios of density between the PSF and β-actin bands. ****p < 0.0001. (**C**) After siPSF was transfected 48 h, virus was incubated of MOI = 0.1. The growth curves of viruses were drawn by assaying the viral titers of the supernatants obtained from 12 h to 72 h post infection by using microtitration infectivity assays. The data from three independent trails are represented as mean ± SD. *t*-test; *p < 0.05.

The PSF protein is associated with various functions, including RNA splicing, viral replication, genetic recombination, and cancer suppression^34–36^. After SX-1 infection, PSF protein expression levels were increased. To clarify the function of PSF in the viral replication cycle, the effect of decreasing the amount of intracellular PSF on the replication of PRRSV was further examined using target-specific RNA interference. Therefore, MARC-145 cells were transfected with PSF siRNA duplexes designed to specifically silence the expression of the PSF gene. As shown in Fig. 5A, MARC-145 cells transfected with PSF siRNA exhibited an approximately 82% decreased level of endogenous PSF protein compared with the cells transfected with scrambled, noneffective siRNAs (Fig. 5B). The reduction in PSF protein concentration resulted in a significant decrease in viral yield through the TCID_50_ quantitative method; particularly, a 12.7-fold decrease at 60 hpi was observed in SiPSF-transfected groups compared with scramble-transfected groups (Fig. 5C). However, no significant effect on the viral yield of the ZCYZ or SD1 strains was noted, indicating that PSF has a different effect on the various virulent strains of PRRSV. Taken together, these results revealed that PSF has a unique upregulated expression and is required for the effective infection of the highly virulent PRRSV SX-1 strain.

## Discussion

Understanding the changes in cellular protein levels after exposure to PRRSV is helpful for elucidating the molecular mechanism associated with functional alterations. We were, in this study, the first to apply an iTRAQ-2D LC–MS/MS method for the proteome profiling of MARC-145 cells infected with PRRSV strains with different virulence. The functional roles of the differentially expressed proteins associated with PRRSV infection are discussed as follows.

In Group I (SX-1 vs. MARC-145), the differentially expressed proteins were mostly involved in morphogenesis, protein synthesis, metabolism, stress response, the receptor complex, and endocytosis. FLNB, FLNA, LMNA, and LMNB1 were supposed to involve an alteration of cytoskeletal networks and cellular communication. HSPD1, HSPA8, HSP90AA1, and HSPE1 were related to stress response^37^. In addition, they acted as molecular chaperones facilitating the assembly of multiprotein complexes, participating in the translocation of polypeptides across cell membranes and to the nucleus, and aiding in the proper folding of nascent polypeptide chains. HSPA8, also known as HSP70, is mainly found in both the cytosol and nucleus of mammalian cells, playing key roles in the cytosolic endoplasmic reticulum and mitochondrial import machinery^38^. The upregulated expression of HSP70 can protect PRRSV-infected MARC-145 cells against apoptosis and be conducive to the replication and spread of the virus, indicating that HSP70 is associated with the pathogenesis of this virus. As a member of the annexin family, ANXA2 is highly expressed in eukaryotic cells and localizes in the cytosol^39^. Precious reports have implicated ANXA2 in the replication of certain viruses^39–41^. Regarding PRRSV, ANXA2 can interact with the PRRSV Nsp9 protein and be incorporated into virions^42^. Importantly, ANXA2 is beneficial for PRRSV replication *in vitro*^43^. The cytoskeleton protein of vimentin (VIM) acts as the receptor complex of PRRSV and forms a complex with Nsp2 by using the viral N protein as an intermediate^44,45^. CLTC (clathrin) is a major protein component of the cytoplasmic face of intracellular organelles, the so-called coated vesicles and coated pits. It has been reported that the entry of PRRSV into cells occurs by specific binding to the outer cell membrane, followed by clathrin-dependent endocytosis. First, the PRRS virions bind to cell-surface receptors; then, they are delivered intact into the endosome through clathrin-coated pits and vesicles. The entire process was demonstrated and proven using confocal microscopy^46–48^.

In Group II (ZCYZ vs. MARC-145), an interesting discovery that differed from other groups was that the expressed proteins, including PSMA1, PSMA2, PSMA7, PSMB4, and PSMD1, were involved in the ubiquitin–proteasome pathway (UPP). This pathway is the major nonlysosomal process responsible for the breakdown of most short-lived and long-lived proteins in mammalian cells. In addition, this pathway controls various major biological events—avoidance of host immune surveillance, viral maturation and viral progeny release, oncogenesis, transcriptional control, signal transduction, receptor downregulation, and antigen processing—via the breakdown of specific proteins^49^. Many viruses have been reported to evolve different strategies to utilize this pathway for their own benefits. For example, the ubiquitin–proteasome system has been suggested to be required for p53 inactivation, apoptosis suppression, viral transcription, and regulation of the human T-cell leukemia virus 1^50,51^. Adenovirus and coronavirus can also make use of the UPP for ubiquitination^52,53^. Recently, the ovarian tumor domain of PRRSV Nsp2 was reported to possess ubiquitin-deconjugating activity, which inhibits NF-κB activation through the prevention of iκBα degradation by interfering with its polyubiquitination process^54^. Nsp11 protein, which has a unique and conserved endoribonuclease, inhibits NF-κB activation by specifically removing lysine 48 (K48)-linked polyubiquitin chains^55^.

In Group III (SD1 vs. MARC-145), only 44 differentially expressed proteins, which was the smallest number among the three groups, were found in the MARC-145 cells infected with a mild virulent SD1 strain. These differentially expressed proteins were mainly related to cell morphology, protein synthesis and metabolism, heat stress response, etc. The central proteins included NCL, DDX3X, FASN, FLNA, HSPA8, and ANXA2. NCL (also called nucleolin) is a type of cellular skeleton protein with the primary function of binding with proteins and nucleic acids. DDX3X is an ATP-dependent RNA helicase that has ATP-binding activity, nucleic acid- and protein-binding activity, and helicase activity. It is mainly involved in species interactions. FASN is mainly related to fatty acid and energy metabolism. FLNA (filament protein A), a skeleton protein in cells, not only has the ability to bind with proteins, nucleic acids, and transcription factors but also possesses the function of signal transduction.

Antisense RNAs, also referred to as natural antisense transcripts or natural regulatory RNAs, are small molecules that mediate regulation and generally inhibit mRNA transcription and/or translation or induce their rapid degradation^13,14^. Signals regarding antisense RNA transcription and regulation were named the antisense pathway, in which PSF and P54 are major proteins^15–17^. After infection with the PRRSV SX-1 strain, PSF and P54 protein expression was upregulated (Fig. 2A), and the antisense pathway was the most variable of all the significant signaling pathways for the highly virulent SX-1 strain; however, this finding was not observed in the signaling pathways for the moderately virulent ZCYZ strain or the mildly virulent SD1 strain (Fig. 2A). Hence, target-specific RNA interference was used to decrease endogenous PSF protein levels to verify its function in PRRSV infection. After decreasing PSF protein concentration, the viral yield of SX-1 significantly decreased, whereas the viral yield of the ZCYZ and SD1 strains were not significantly affected (Fig. 5), indicating that PSF protein has different functions in various virulent PRRSV strains. As a major protein of the antisense pathway, the silencing of PSF expression is expected to affect antisense pathway function. Accordingly, the molecular mechanism of PSF and antisense pathway function in the infection of different virulent PRRSV strains will be assessed in further research.

## Conclusions

We displayed the “colorful” proteome profile of MARC-145 cells infected with different PRRSV strains using the iTRAQ coupled with 2D LC–MS/MS approach for the first time. A total of 233 significantly altered proteins were identified, and we proved that PSF protein has different functions in various virulent PRRSV strains. The study provided an abundance of useful information to study the diversification of MARC-145 cells infected with different PRRSV strains, and these data would help to understand the interactions between this virus and its host.

## Electronic supplementary material

Supplementary information: Original WB data
