# Supplementary material for: Porcine Reproductive and Respiratory Syndrome Virus strains with Higher Virulence Cause Marked Protein Profile Changes in MARC-145 Cells
Source: Sci Rep. 2018 Oct 9;8:15000. doi: 10.1038/s41598-018-32984-0 (PMC6177479; doi:10.1038/s41598-018-32984-0)

Porcine Reproductive and Respiratory Syndrome Virus strains with Higher Virulence Cause Marked Protein Profile Changes in MARC-145 Cells

Zhi Chen [a, 1], Shaoning Liu [c, 1], Shujin Zhang [d], Yuyu Zhang [a, b], Jiang Yu [a, b], Wenbo Sun [a], Lei Chen [a], Yijun Du [a], Jinbao Wang [a], Yubao Li [d], Jiaqiang Wu [a, b, *]

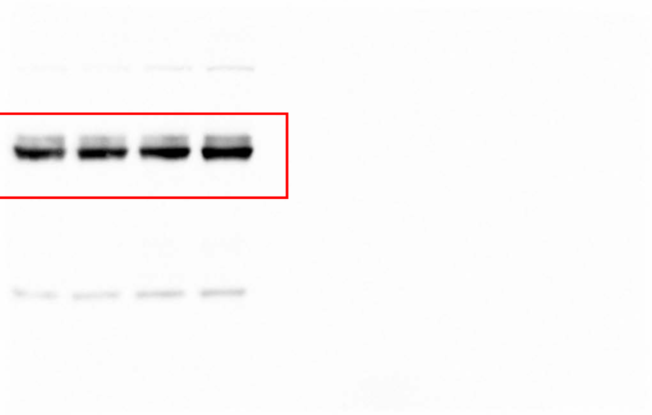

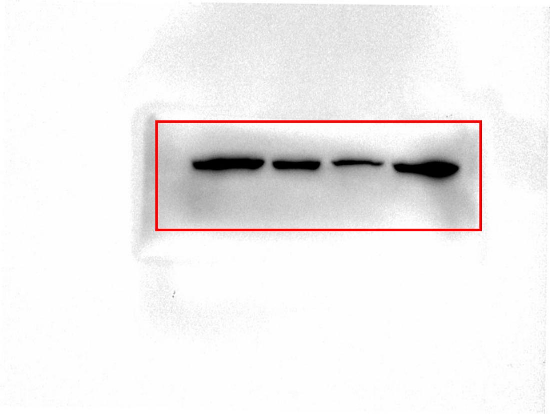

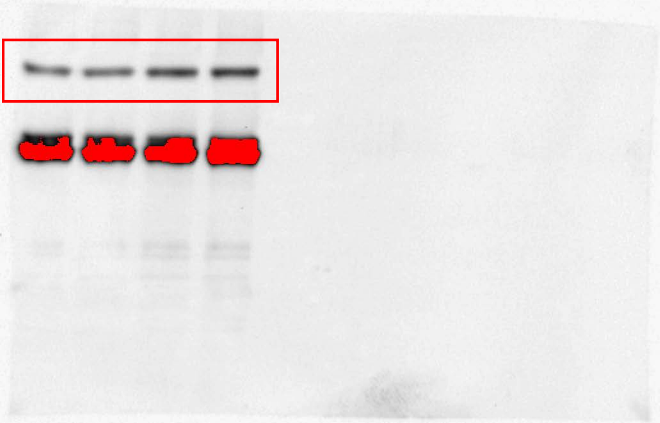

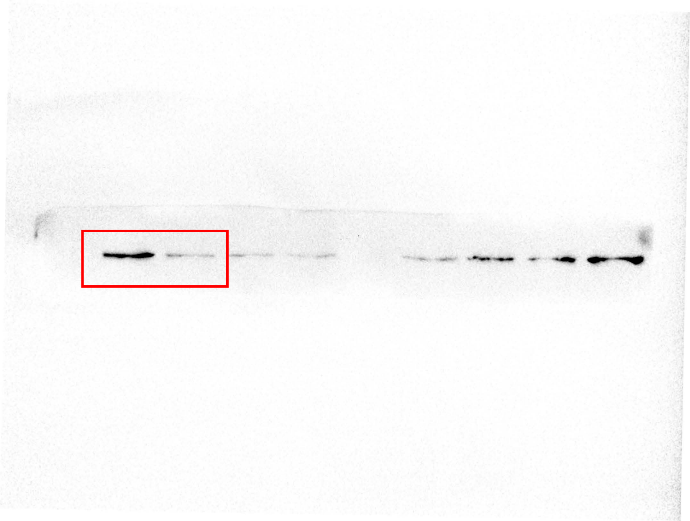

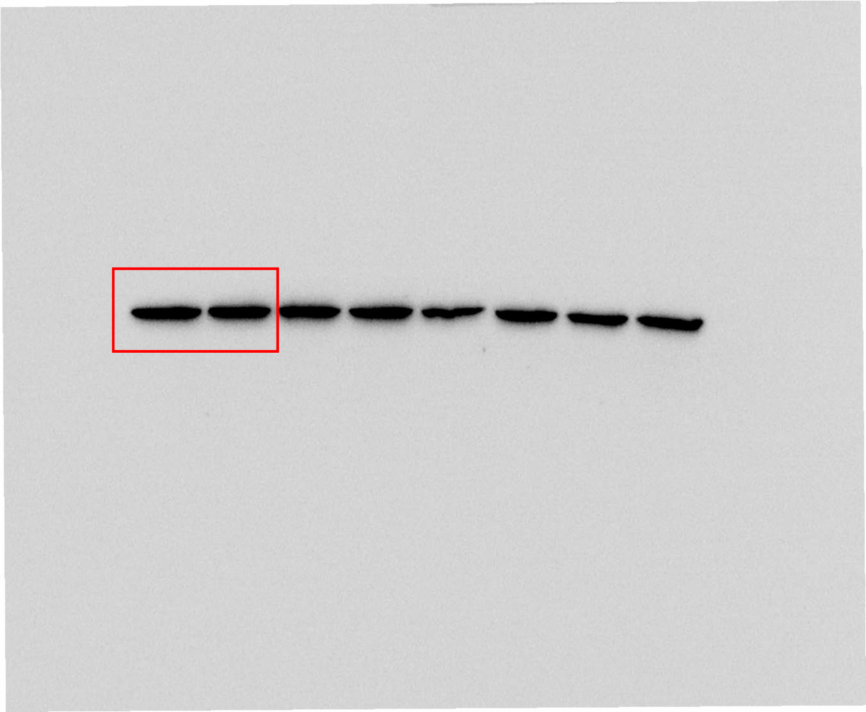

Supplement: Supplementary file 1 — Supplementary information: Original WB data [file 41598_2018_32984_MOESM1_ESM.pdf]
